# Uncovering the Risks and Benefits of Patent Foramen Ovale (PFO) Closure in Cryptogenic Stroke: A Case Study

**DOI:** 10.7759/cureus.70595

**Published:** 2024-10-01

**Authors:** Matthew P Bommarito, Great Ogujiofor, Neha Patel, Rami Akel, Marek Krysztofiak

**Affiliations:** 1 Internal Medicine, Hospital Corporation of America (HCA) Florida Bayonet Point Hospital, Hudson, USA; 2 Cardiology, Hospital Corporation of America (HCA) Florida Bayonet Point Hospital, Hudson, USA

**Keywords:** atrial fibrillation (afib), cerebral vascular accident, cryptogenic strokes, patent foramen ovale (pfo), percutaneous patent foramen ovale repair

## Abstract

Patent foramen ovale (PFO) exists in 20-25% of the adult population, and about 40% of adults who present with cryptogenic stroke (CS) have a PFO. The benefit of intervention has been debated with regard to stroke prevention given the high risk of postoperative atrial fibrillation (AF). In light of this, clinical decision-making is guided by PFO-Associated Stroke Causal Likelihood (PASCAL) classification and Risk of Paradoxical Embolism (RoPE) score analysis. Contemporary studies have shown superlative benefits of PFO with device closure and antiplatelet therapy. In our case study, we describe a 52-year-old female with no comorbid conditions who initially presented with expressive aphasia. Upon further diagnostic workup, she was found to have a PFO on transesophageal echocardiogram (TEE). Cardiac monitoring revealed no underlying arrhythmias. The patient subsequently underwent successful PFO repair with device-assisted closure and intracardiac monitor placement. She was discharged with dual antiplatelet therapy. On postoperative follow-up, no detected arrhythmias were revealed, and she made a meaningful recovery with near-total resolution of her symptoms. Our case raises the question of the extent of comprehensive evaluation to rule out underlying etiologies including AF prior to considering device-assisted closure and suggests the need for further studies.

## Introduction

Patent foramen ovale (PFO) represents a common congenital heart anomaly characterized by the persistence of an opening between the atria, allowing flow between them [1]. While often asymptomatic, PFO has been implicated in various clinical conditions, including cryptogenic stroke and paradoxical embolisms. PFO closure has emerged as a potential therapeutic option to mitigate associated risks but its benefit has been debated [2-6]. Suboptimal patient selection was thought to have been attributed to prior studies, which consequently led to a comprehensive analysis of randomized control trials that studied a more representative population. Due to this, concerns regarding discrepant findings were addressed, and the application of benefits to clinical use yielded superlative benefits.

Of note, there remains uncertainty of preoperative and postoperative observation for underlying and acquired arrhythmias, respectively, after device closure. There are well-documented risks that come with this intervention, and patient selection is endorsed to be chosen carefully. Atrial fibrillation (AF) remains one of the most common postoperative concerns. In this case study, we shed light on candidates who may benefit from PFO-device closure and urge the need for comprehensive evaluation including cardiac monitoring before and after intervention.

In this case report, we highlight a PFO device closure, as well as the patient's clinical background, the rationale behind PFO closure, and the subsequent outcome. Through the meticulous documentation of this case, we aim to contribute valuable insights to the growing body of evidence surrounding PFO closure, fostering a deeper understanding of medical versus interventional therapy and post-procedure monitoring.

## Case presentation

**Figure 1:**
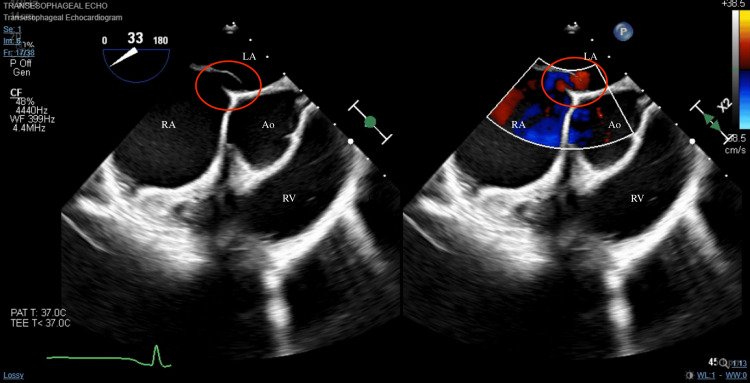
Transesophageal echocardiogram bicaval view depicting the patent foramen ovale Left: Inflow-outflow non-color doppler transesophageal echocardiogram bicaval view revealing patent foramen ovale (red circle) between the right atrium (RA) and the left atrium (LA). Aorta depicted for reference (Ao). Right: Inflow-outflow color doppler transesophageal echocardiogram bicaval view demonstrating bilateral flow of the patent foramen ovale (red circle) between the right atrium (RA) and the left atrium (LA). Aorta depicted for reference (Ao).

**Figure 2:**
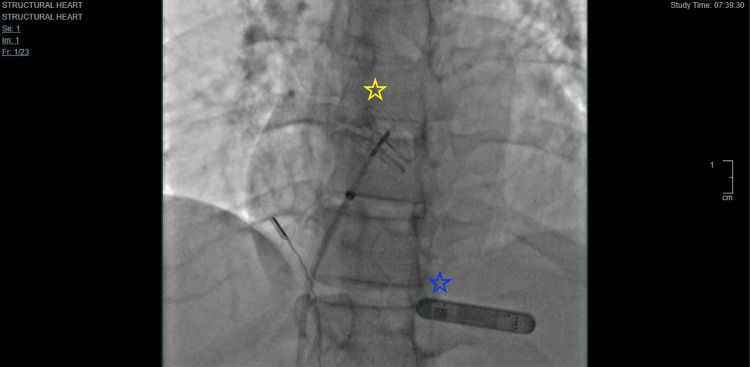
Angiographic view of patent foramen ovale occluder and insertable cardiac monitor Yellow star: 25 mm occluder located between the left and right atrium. Blue star: Insertable cardiac monitor remaining in adequate place.

A 52-year-old female presented as a transfer to our facility for isolated receptive and expressive aphasia. The patient worked in law enforcement, actively traveled, and was training for recreational distance running. The patient denied the need for prescribed medications and also denied a personal or family history of arrhythmias, stroke, blood clots, coronary artery disease, hypertension, or diabetes. Her symptoms started the night before admission with a limited verbal response, which progressed to worsening expressive aphasia the next morning. Her vitals on admission were stable but the patient’s verbal response was limited to the word “yes” to open-ended questions and also displayed receptive aphasia. Compute tomography (CT) head revealed an acute infarct in the left insula. Magnetic resonance imaging (MRI) of the brain confirmed the involvement of the left middle cerebral artery territory. Given the patient’s low National Institutes of Health Stroke Scale (NIHSS) score of 7 and low area of occlusion, the risks of mechanical thrombectomy outweighed the benefits. Moreover, the patient was outside the appropriate window for fibrinolytic therapy. Thus, she was initiated on a high-intensity statin, aspirin 81 mg daily, and clopidogrel 75 mg daily. Extensive lab work, including complete blood count, metabolic panel, coagulation panel, lipid panel, hemoglobin A1c, thyroid panel, and autoimmune/hypercoagulable panel tests were unremarkable. Carotid dopplers, lower extremity venous duplex, and transthoracic echocardiogram (TTE) were also unremarkable. Telemetry was significant for sinus bradycardia with a heart rate in the 50s. During her six days of hospitalization, she demonstrated marked improvement in aphasia with subsequent speech therapy visits. A transesophageal echocardiogram (TEE) with agitated saline revealed a bidirectional, predominantly left-to-right PFO on color and bubble study with no significant valvular abnormalities and no left atrial appendage thrombus (Figure 1). This was confirmed with a duplex evaluation of the right internal carotid artery which detected six microbubbles on normal respiration and nine microbubbles following a Valsalva maneuver. The patient had an insertable cardiac monitor (ICM) recorder placed and subsequently underwent fluroscopy-guided PFO closure (Figure 2). She tolerated both procedures well and with no complications. Telemetry monitoring revealed sinus rhythm and repeated TTE revealed preserved left ventricular function with closure device in place and an absence of pericardial effusion. The patient was discharged on dual antiplatelet therapy. She was evaluated on an outpatient basis six months postoperatively and did not experience any arrhythmias, including AF. Her expressive aphasia had substantially improved, and she did not suffer any further recurrence of stroke.

## Discussion

Most patients with a PFO are asymptomatic, however, the association with cardiac and medical manifestations is well documented. Given the high prevalence of PFO in the general population and the low risk of stroke related to PFO, there has been a degree of uncertainty about the causal relationship between a PFO and an embolic-appearing ischemic stroke with no other evident stroke mechanism despite a comprehensive evaluation [2]. The Northern Manhattan Study (NOMAS) was a multiethnic prospective cohort of Northern Manhattan which revealed that approximately one-third of PFOs detected were incidental findings and not associated with an increased risk of stroke [3]. A population-based cohort study aimed to evaluate the clinical effectiveness of PFO closure and found no statistically significant association between the risk of cryptogenic stroke (CS) and the presence of a PFO [4]. Petty et al. pointed out in a population-based study that the generalized population in previous studies did not showcase a representative patient selection [5]. The discrepant findings are likely to be explained by the PFO-attributable fraction of stroke. It was discerned that the study's limited number of patients under age 55 resulted in wide confidence intervals in the analysis of that subgroup. Consequently, the study did not disprove the argument that a PFO is linked to a heightened risk of ischemic stroke in children or young adults. In light of this, a case-control trial revealed that PFO was prevalent in CS, particularly in younger patients (age <60) [6]. This called for further objective analysis, and different possibilities that stroke was causally related to PFO were ultimately suggested by the Risk of Paradoxical Embolism (RoPE) score and modified PFO-Associated Stroke Causal Likelihood (PASCAL) classification [7]. These tools can be used to estimate the probability of whether a PFO is associated with CS as it analyzes parameters such as age, risk factors, shunt size, imaging results, etc. To generalize, younger patients (age <60) with no history of hypertension, diabetes, stroke, or transient ischemic attacks would have a high RoPE score and PASCAL classification, raising clinical suspension for PFO-associated stroke. Further management should be individualized for these patients and medical therapy with or without potential percutaneous intervention should be considered.

Standard pharmacologic therapy for stroke prevention in PFO involves antiplatelet or anticoagulation therapy. In comparative studies, there were no significant differences in efficacy between anticoagulants and antiplatelet therapy in PFO-associated stroke. The PFO in Cryptogenic Stroke Study (PICSS) sought to compare aspirin and warfarin in a double-blind study analyzing rate of recurrent ischemia or death, which revealed no significant difference [8]. The PFO Closure or Anticoagulants versus Antiplatelet Therapy to Prevent Stroke Recurrence (CLOSE) trial demonstrated the rate of recurrent ischemic stroke after PFO closure is increased without antiplatelet therapy compared with antiplatelet therapy [9]. Additionally, anticoagulated patients in these trials experienced more frequent minor and nominally more major bleeding complications in stroke prevention. A meta-analysis of 12 prospective observational studies and randomized trials also suggested that anticoagulation was not significantly more effective than antiplatelet therapy [10].

Clinical trials have shown that PFO device closure combined with long-term medical antiplatelet therapy significantly reduces the recurrence of ischemic stroke compared to long-term medical antithrombotic therapy alone (antiplatelet or anticoagulant). A meta-analysis demonstrated that device closure reduced the rate of recurrent ischemic stroke with a hazard ratio (HR) of 0.30 (95% CI, 0.13-0.68; P=0.004) compared to medical therapy alone​ [11]. Note that pursuing intervention of PFO closure with a device comes with risks, and candidates who are considered for this operation should be selected carefully. Patients undergoing PFO closure for secondary stroke prevention were reported to have an adverse outcome in 8.9% of subjects analyzed, including results that were reported in Randomized Evaluation of Recurrent Stroke Comparing PFO Closure to Established Current Standard of Care Treatment (RESPECT), Evaluation of the STARFlex Septal Closure System in Patients with a Stroke and/or Transient Ischemic Attack due to Presumed Paradoxical Embolism Through a PFO (CLOSURE I), and Percutaneous Closure of PFO Using the Amplatzer PFO Occluder with Medical Treatment in Patients with Cryptogenic Embolism (PC) trials [12]. Additionally, when considering the risk for PFO closure, it was reported that new-onset AF was found to be more frequent in patients who underwent PFO closure in comparison to antithrombotic therapy (RR=4.33, 95% CI, 2.37-7.89; I^2^=14%) [13]. 

The Society of Cardiovascular Angiography and Intervention (SCAI) has provided contemporary guidelines for PFO management as an evidence-based collection of recommendations supported by data pooled from retrospective studies mentioned above. The SCAI guidelines address patient selection and outline current recommendations to support PFO closure in PFO-associated stroke [14]. Specific to our patient, PFO closure rather than antiplatelet therapy alone was favored given her high RoPE score and absence of other embolic etiologies. These benefits of PFO closure greatly offset the risk of transient AF secondary to device closure; however, AF risk remains a valid concern that warrants long-term monitoring. 

In one particular study, the Cryptogenic Stroke and Underlying AF (CRYSTAL-AF), a randomized control study conducted on 441 patients, the authors assessed whether long-term monitoring in patients with an intracardiac monitor after CS was better than standard follow-up for detecting AF. These authors found that continuous monitoring with an intracardiac monitor (at six months and 12 months) significantly increased the detection rate of AF compared to conventional follow-up methods, suggesting that this approach could improve stroke prevention strategies in high-risk patients [15]. Limitations of this study in scope provided that future analysis should ensure adequate exclusion of baseline AF with cardiac monitoring and utilize close AF follow-up to determine the true incidence of AF.

There are few studies in which the possibility of pre-existing AF prior to PFO closure has been considered. No clear guidelines currently exist for intracardiac monitoring before device closure. Our case demonstrates that AF did not emerge during the patient's hospitalization leading up to device closure and did not appear more than six months postoperatively, suggesting that our patient may not have had underlying AF and tolerated the device closure well.

## Conclusions

We reported an exemplary case of a 52-year-old patient who was found to have a PFO-associated stroke and underwent device closure without difficulty. She has made substantial recovery without recurrence of stroke or arrhythmias. The case highlights the importance of identifying candidates who may benefit from interventional therapy and describes literature that supports this. We draw attention to the need for additional studies to exclude preoperative and postoperative arrhythmias prior to PFO closure, including AF.
